# Dual role of microorganisms in metal corrosion: a review of mechanisms of corrosion promotion and inhibition

**DOI:** 10.3389/fmicb.2025.1552103

**Published:** 2025-04-09

**Authors:** Qianwei Li, Lingli Gong, Xiaoji Chen, Geoffrey Michael Gadd, Daoqing Liu

**Affiliations:** ^1^State Key Laboratory of Petroleum Pollution Control, China University of Petroleum, Beijing, China; ^2^Geomicrobiology Group, School of Life Sciences, University of Dundee, Dundee, United Kingdom

**Keywords:** microbiologically-influenced corrosion, microbially-influenced corrosion inhibition, corrosion mechanisms, biomineralization, biofilm

## Abstract

The dual role of microorganisms in metal corrosion and corrosion inhibition reflects their complex biochemical interactions. In terms of corrosion, certain microorganisms accelerate metal oxidation by producing acidic metabolites or facilitating electrochemical processes, thereby causing damage to the material. Conversely, under specific conditions, they can form biofilms and/or biominerals that create protective layers, reducing the oxidation rate and delaying corrosion. This paper provides a comprehensive illustration of microbial corrosion promotion and inhibition, emphasizing the importance of key microorganisms involved in these corrosive processes. Microorganisms, including sulfate-reducing bacteria, nitrate-reducing bacteria, iron-oxidizing and iron-reducing bacteria and certain fungi, contribute to corrosion through their metabolic activities. Microbial corrosion mechanisms can be classified into extracellular electron transfer, microbial metabolism corrosion and the oxygen concentration cell theory. In contrast, microorganisms can effectively mitigate metal corrosion through a range of mechanisms including reduction of dissolved oxygen levels, secretion of antimicrobial substances, biological competition and biomineralization. Microbial corrosion and inhibition generally arise from multiple mechanisms working together, rather than a single cause. A deeper understanding of these mechanisms can provide a theoretical basis and practical guidance for the development of new anti-corrosion strategies.

## Graphical abstract

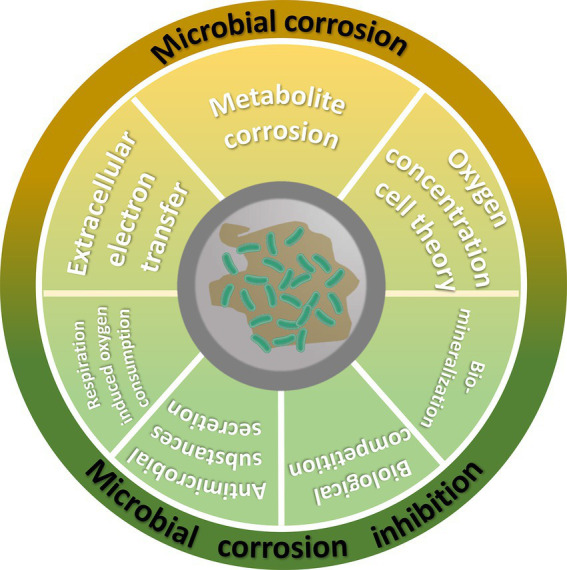

## 1 Introduction

Corrosion is a global industrial risk and corrosion-related consequences are estimated to cost $2.5 trillion a year for inspection, correction, and prevention (Lakshman et al., 2024). It has been reported that China’s annual economic cost for materials corrosion accounts for 3.4 to 5.0% of GDP, with a total loss of more than 400 billion yuan (Hou et al., 2017).

Microorganisms, as the most prosperous life on Earth, are widely distributed in natural environment and associated with all human activities. They play key roles in biogeochemical cycles, environmental and ecosystem health. In the area of materials science, the influence of microorganisms cannot be ignored, especially in corrosion and corrosion inhibition, where the roles of microorganisms are complex and multifaceted (Kip and Van Veen, 2015). Microbial corrosion, commonly referred to as microbiologically or microbially-influenced corrosion (MIC), refers to corrosion resulted from microorganisms (including bacteria, archaea, fungi, etc.) and their metabolites exhibiting direct or indirect corrosive effects on metals (Unsal et al., 2016). This can also be called microbial corrosion, bacterial or fungal corrosion, or biological corrosion (biocorrosion) (Loto, 2017). Microorganisms can both accelerate or slow down materials corrosion to varying extents (Yu et al., 2023). Corrosion inhibition by microorganisms may involve biological competition, inhibition of microbial attachment, secretion of antibiotic substances and the formation of biominerals (Wang et al., 2022).

Microbial corrosion can be found in widely different scenarios. In the complex soil environment, buried pipelines will suffer from several types of corrosion factors (Liu and Cheng, 2020) and microorganisms participate in several such corrosion processes. Microorganisms, such as bacteria, fungi, archaea, and other microorganisms in marine environments, have been found to accelerate corrosion. Some of them can produce organic acids during growth which accelerates the corrosion degree of carbon steel, Mg and Al alloy materials (Shan et al., 2024). In oil and gas field pipelines, carbon steel is widespread use in engineering material, but is prone to abiotic and biological corrosion damage (Madirisha et al., 2022). It has been found that gradually increasing the dissolved organic carbon (DOC) from zero to 3.0 ppm, could enhance the growth of archaea and promote a cathodic response by consuming more of the metal iron as an energy source and therefore intensifying local corrosion. When the DOC was increased to 5.0 ppm, the biofilm appeared evenly and the corrosion phenomenon was uniform corrosion (Qian et al., 2019).

Microorganisms also show considerable potential for corrosion inhibition. Some microorganisms can produce bioactive substances with corrosion inhibition effects, such as antioxidants, alkaloids and organic acids (Wang et al., 2022). These substances can form a stable protective film on metal surface, slowing down or preventing corrosion reactions. Microorganisms can also build a physical barrier to protect metals from corrosive environments by the formation of biofilms and secretion of extracellular polymeric substance (EPS). Victoria et al. (2021) proposed that biofilms formed by bacteria could inhibit or accelerate biological corrosion of metals depending on local environmental conditions. In addition, biomineralization processes can play an important role in inhibiting corrosion. By utilizing biomineralization activities of microorganisms, a dense biomineralized film can be formed on the surface of materials such as metals or concrete. Biomineralization of calcite induced by *Bacillus subtilis* was applied to reduce the corrosion degree of steel in seawater, and the biomineralized film was composed of EPS and calcite, showing stable anti-corrosion activity (Guo et al., 2019).

In-depth research on the mechanisms of corrosion microorganisms and corrosion inhibition will not only enable understanding of the causes and hazards of microbial corrosion more comprehensively, but also provide theoretical support and practical guidance for the development of efficient and environmentally-friendly anti-corrosion technologies and materials. With the continuous development of biotechnology and materials science, the prospect of microbial application in the field of corrosion control will become broader.

## 2 Microorganisms involved in corrosion

### 2.1 Sulfate-reducing bacteria

Sulfate-reducing bacteria (SRB) are a group of microorganisms capable of reducing sulfate to sulfide in anaerobic environments (Nofal et al., 2022). They are among the most significant contributors to microbial corrosion. These organisms obtain energy by oxidizing organic compounds or molecular hydrogen, while reducing sulfate, sulfite, thiosulfate, or elemental sulfur as the final electron acceptor in their electron transport chain, producing hydrogen sulfide (H₂S) as a byproduct. To date, over 220 species of SRB have been identified across more than 60 genera. Equations 1, 2 illustrate the use of sulfate as the terminal electron acceptor, providing insight into the energetics of microbial-induced corrosion (MIC) caused by SRB (Anusha and Mulky, 2023).


(1) $$\text{Anodic}:Fe\to {Fe}^{2+}+2e^{-}$$

(2) $$\text{Cathodic}:{\text{SO}}_{4}^{2-}+9H^{+}+8e^{-}\to {HS}^{-}+4H_{2}O$$

Sulfate-reducing bacteria (SRB) are found in various environments and are particularly prevalent in oil and gas field operations. Among the numerous types of corrosive bacteria present, SRB are the most significant. The lubricating fluids and organic matter within the collection and transportation systems in these industries serve to provide essential nutrients, fostering the proliferation of bacteria. As a result, large populations of SRB and other corrosive bacteria accumulate in pipeline networks, leading to significant pipeline corrosion (Ibrahim et al., 2018). In marine environments, sulfate-reducing bacteria (SRB) can have a significant impact on the corrosion rate of metals. Long et al. (2018) investigated the corrosion behavior of Zn-Al-Mg-RE coatings in seawater containing SRB, and demonstrated that the corrosion rate initially increased before gradually slowing down. In the early stages of immersion, SRB metabolites were the primary drivers of coating corrosion. However, in the middle to later stages, the reaction between corrosion products and bacterial metabolites formed ZnS which filled the pores and partially suppressed further corrosion. Soil harbors various corrosive bacteria, with SRB being particularly prominent. Sani et al. (2016) conducted studies on the combined effects of SRB, soil type, and water content on the corrosion behavior of pipeline steel. The findings revealed that the presence of SRB in soil accelerated the corrosion rate and intensified pitting. However, under conditions of high water content, SRB exhibited a protective effect.

### 2.2 Nitrate-reducing bacteria

Nitrate-reducing bacteria (NRB) can utilize electrons released during iron oxidation, using intracellular nitrate as the terminal electron acceptor. They reduce nitrate to nitrite and may further process nitrite into gaseous nitrogen compounds or convert it into NH₄^+^ through denitrification (Equations 3, 4; Liu et al., 2021).


(3) $$\text{Oxidation reaction}:Fe\to {Fe}^{2+}+2e^{-}$$

(4) $$\text{Reduction reaction}:{\text{NO}}_{3}^{-}+8e^{-}+10H^{+}\to {NH}_{4}^{+}+3H_{2}O$$

When SRB and NRB coexist, NRB have been found to inhibit SRB-induced corrosion through two primary mechanisms (Sun et al., 2024). The first mechanism is biological competitive exclusion, where NRB outcompete SRB for organic carbon sources, thereby inhibiting SRB growth (Fu et al., 2022). The second mechanism involves metabolite inhibition, where the metabolic byproducts of NRB suppress the activity of SRB (Lai et al., 2020). Adding nitrate activates NRB, promoting their growth while inhibiting SRB. This inhibition reduces hydrogen sulfide production by SRB, effectively alleviating reservoir acidification in oil and gas fields (Xu et al., 2013). In anoxic environments, NRB obtain electrons by reducing nitrate and iron loses electrons and forms iron oxides leading to microbial corrosion. Liu et al. (2021) found that NRB accelerated uniform and pitting corrosion of X80 steel. Yuk et al. (2020) isolated the nitrate-reducing bacterium *Marinobacter* YB03, which was unable to mitigate acidification caused by the sulfate-reducing bacterium *Desulfotignum* YB01. They examined the influence of YB03 on acidification and microbiologically-influenced corrosion, discovering significant pitting corrosion on carbon steel samples associated with YB03. These results confirmed the strain’s role in exacerbating both acidification and corrosion.

### 2.3 Iron-oxidizing bacteria

Iron-oxidizing bacteria (IOB), also known as iron bacteria, were first described by Winogradsky in 1888. Iron-oxidizing bacteria (IOB) oxidize ferrous iron to ferric iron through aerobic respiration, releasing energy in the process. This energy is used by IOB to meet their needs for growth and reproduction. This process changes the balance of iron oxidation reactions leading to the generation of oxygen concentration difference batteries on the metal surface causing local corrosion, and eventually forming ferric hydroxide (Fe(OH)_3_) precipitates (Starosvetsky et al., 2001; Duan et al., 2010; Wang et al., 2014).

### 2.4 Iron-reducing bacteria

Iron-reducing bacteria (IRB) are microorganisms capable of reducing ferric ions (Fe^3+^) to ferrous ions (Fe^2+^). These bacteria can be strictly anaerobic or facultatively anaerobic. There are many reports of microbially-induced biomineralization where reduction of mineral Fe^3+^ to soluble Fe^2+^ results in subsequent combination with other ions in the system to form new minerals. At the same time, Fe^2+^ can further react with the remaining Fe^3+^ minerals to form mixed Fe^2+^/Fe^3+^ minerals. There is growing attention given to the corrosive effects of iron-reducing bacteria on metal materials. Jamaluddin et al. (2022) reported that certain iron-reducing bacteria (IRB), such as *Shewanella oneidensis* MR-1, *Shewanella chilikensis*, and *Geobacter sulfurreducens*, contribute significantly to metal corrosion. These bacteria participate in the corrosion processes due to their ability to reduce Fe^3+^ by their electron transport chains. This reduction provides energy and leads to pitting of steel resulting in severe corrosion. Conversely, it has been demonstrated that certain iron-reducing bacteria can play a role in inhibiting corrosion. Lee et al. (2006) investigated the effects of biofilms formed by *Shewanella oneidensis* (MR-1) and *Desulfovibrio desulfuricans* (G20) on carbon steel corrosion, and it was found that co-cultures of MR-1 and G20 were able to protect the steel from corrosion over a short period. This indicates that iron-reducing bacteria may function as corrosion inhibitors, even in the presence of bacteria that promote corrosion.

### 2.5 Fungi

Fungi are eukaryotic microorganisms that, despite being less frequently studied, can still have corrosive effects. Dai et al. (2016) investigated the corrosive effects of *Aspergillus niger* on aluminum alloy (AA 2024) and found that these fungi significantly accelerated the corrosion. This corrosion was primarily driven by oxalic acid production by *A. niger* which resulted in the formation of micropits and extensive pitting. Ching et al. (2016) isolated a novel basidiomycete, *Moniliella wahieum* Y12T, from a 20% biodiesel mixture, and it was found to cause corrosion of steel sheets, with enhanced corrosion observed on the metal surface when the medium was acidified. Table 1 provides some information about microorganisms causing microbial corrosion.

**Table 1:** Microorganisms causing microbial corrosion.

Microorganisms Type	Name	Origin	Corrosion effect	References
NRB	*Pseudomonas aeruginosa*	Drinking water	Corrosive pitting	Jia et al. (2017)
	*Bacillus licheniformis*	Soil	Corrosion of C1018 carbon steel	Xu et al. (2013)
	*Klebsiella mobilis*	Marine	Corrosion pits on the surface of C1018 carbon steel	Etique et al. (2014)
	*Marinobacter*	Marine	Severe pitting corrosion on carbon steel	Yuk et al. (2020)
SRB	*Desulfovibrio vulgaris*	Anaerobic soil	–	Dou et al. (2019)
	*Desulfovibrio desulfuricans*	Soil	Severe anodic steel dissolution	Eduok et al. (2019)
	*Archaeoglobus fulgidus*	Marine	Corrosive to C1018 carbon steel	Jia et al. (2018)
	*Pseudomonas aeruginosa*	Marine	Corrosion to stainless steel	Huang et al. (2018)
IOB	*Acidithiobacillus ferrooxidans*	–	Accelerates the corrosion of C1010 carbon steel	Mustin et al. (1992)
	*Thiobacillus denitrificans*	–	Metal corrosion	Baker and Banfield (2003)
	*Leptothrix*, *Crenothrix*, *Sphaerotilus*	Estuarine and Marine, Coastal site	–	Weber et al. (2006) and McBeth and Emerson (2016)
IRB	*Sediminibacterium* sp., *Shewanella chilikensis*, *Geobacter sulfurreducens*	Steel pipe	Severe pitting of steel	Jamaluddin et al. (2022)
Fungi	*Aspergillus niger*	–	Corrosion of aluminum alloy	Dai et al. (2016)
	*Aspergillus terreus*	Marine	Corrosion of aluminum alloy	He et al. (2022)

## 3 Microbial corrosion mechanisms

Microbial corrosion mechanisms can be broadly classified into three main categories. One such mechanism, extracellular electron transfer (EET-MIC), involves corrosion caused by the direct transfer of electrons between microorganisms and metals. Metabolite-induced corrosion (M-MIC) refers to corrosion resulting from the action of corrosive metabolites produced by microorganisms. Oxygen Concentration Cell Corrosion refers to localized corrosion occurring due to the establishment of oxygen concentration cells during microbial growth and metabolism of microorganisms.

### 3.1 Extracellular electron transfer mechanism

The classical theory of cathodic depolarization suggests that in a corrosive system, iron oxidation occurs at the anode, while hydrogen evolution reaction occurs at the cathode. Sulfate-reducing bacteria (SRB) can accelerate the dissolution of iron (Fe) by reducing sulfate ions (SO₄^2−^) to hydrogen sulfide (H₂S), which consumes the hydrogen produced on the metal surface (Daniels et al., 1987). However, it has been discovered that SRB can bypass the use of hydrogen (H₂) as an electron donor, directly utilizing the metal to obtain electrons for sulfate reduction. This behavior challenges the classical theory of cathodic depolarization which fails to fully explain the corrosive effects of these bacteria. Accordingly, Enning et al. (2012) introduced the concept of electrical microbially influenced corrosion (EMIC), which suggests that bacteria can directly or indirectly harness electrons from the metal surface for their growth and metabolism, resulting in metal corrosion. This specific form of microbial corrosion is classified as extracellular electron transfer microbially influenced corrosion (EET-MIC).

**Figure 1:**
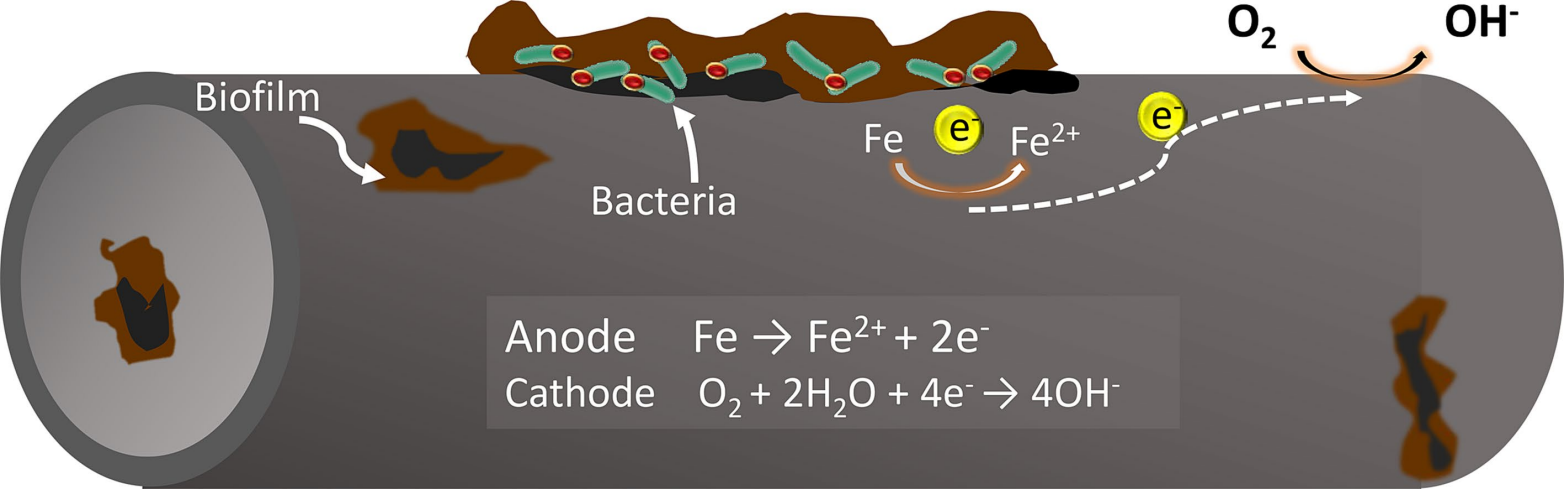
Mechanisms of electron transfer from metal to cell surface in SRB: (a) bacterial outer membrane in direct contact with the metallic surface; (b) electron transfer mediated by redox-active molecules through oxidation and reduction cycles.

Extracellular electron transfer (EET) is typically categorized into two types: Direct Electron Transfer (DET) and Mediated Electron Transfer (MET) (Figure 1). Certain microorganisms, which possess redox-active proteins on their extracellular membranes, can act as electron carriers, transferring extracellular electrons directly to the intracellular environment. This process accelerates cathodic reactions and promotes metal corrosion. For instance, the sulfate-reducing bacterium *Desulfovibrio ferrophilus* IS5 can transfer extracellular electrons directly into the cytoplasm via outer membrane cytochrome c (Omc) or through type IV pili attached to the Omc surface. This mechanism is known as Direct Electron Transfer (DET) (Deng et al., 2018). Unlike direct electron transfer, which requires microbial functional proteins to be in direct contact with the metals, indirect electron transfer is facilitated by electron transfer mediators (ETMs) such as riboflavin and flavin adenine dinucleotides. These mediators can greatly enhance the efficiency of microbial extracellular electron transfer (Chiranjeevi et al., 2019; Zhao et al., 2021).

### 3.2 Metabolite corrosion mechanism

Inorganic metabolites, such as H₂S, produced by the metabolism of sulfate-reducing bacteria (SRB), can acidify the pipe wall environment, thereby accelerating electrochemical corrosion. Additionally, H₂ generated from H₂S dissolved in water can cause hydrogen embrittlement of the metal resulting in crevice corrosion (Raman et al., 2013). The formation of FeS from the reaction of the SRB metabolite sulfide and Fe^2+^ facilitates electron transfer and also exacerbates corrosion.

Organic acids secreted as a result of microbial metabolic activities are a major cause of metal corrosion. These acids are significantly more corrosive than sulfuric and other strong acids at the same pH, due to their buffering capacity and ability to generate additional protons (Kryachko and Hemmingsen, 2017). Acid-producing bacteria (APB) are primarily fermentative microorganisms that can produce significant amounts of acetic acid, formic acid, HNO₃, and H₂SO₄ in both aerobic and anaerobic environments. These organisms acidify their surroundings, causing metal corrosion, severe pitting, and pore leakage (Jia et al., 2019).

### 3.3 Oxygen concentration cell theory

**Figure 2:**
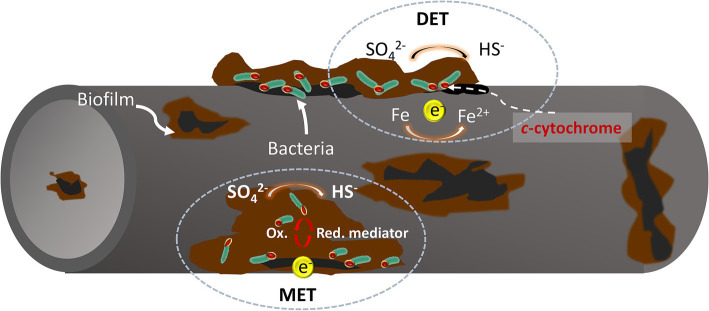
Metal pitting caused by oxygen concentration cells formed by oxygen depletion under a biofilm. Extracellular electron transfer (EET) is typically categorized into two types: Direct Electron Transfer (DET) and Mediated Electron Transfer (MET).

There are three primary mechanisms for generating oxygen concentration cells on metal surfaces (Figure 2). Firstly, heterogeneous distribution of microbial cells can lead to uneven oxygen diffusion across the metal surface. Secondly, the partial accumulation of corrosion products can create localized oxygen depletion. Thirdly, variations in oxygen consumption through aerobic bacterial respiration in different areas can also contribute. In this scenario, the oxygen-poor region acts as the anode and the oxygen-rich region acts as the cathode, leading to localized corrosion due to these oxygen concentration differences. This phenomenon is known as an oxygen concentration difference cell corrosion (Enning et al., 2012). Biofilm of the oxygen-demanding bacterium *Pseudomonas aeruginosa*, can accelerate metal pitting by creating anodic and cathodic regions with varying oxygen concentrations (Abdolahi et al., 2015).

## 4 Mechanisms of microbiologically-influenced corrosion inhibition

Microorganisms can effectively mitigate metal corrosion through a range of mechanisms, including biological expulsion, the secretion of corrosion inhibitors, the production of extracellular polymers, the reduction of dissolved oxygen levels, the formation of biofilm barriers, the secretion of biosurfactants, and the establishment of non-biofilm barriers. Microbial inhibition of metal corrosion typically arises from the synergistic interaction of multiple mechanisms rather than being attributed to a single process. Employing microorganisms for corrosion inhibition offers significant advantages, including self-regulation, in-situ repair, and intelligent protective strategies, thereby potentially providing long-lasting and environmentally sustainable solutions. A thorough understanding of the underlying mechanisms of microbial corrosion inhibition is essential for developing effective metal protection strategies. Furthermore, identifying the challenges and limitations inherent in the research and application of microbial anti-corrosion technologies will inform the direction of future advances in this field.

### 4.1 Bacterial respiration-induced oxygen consumption inhibition of metal corrosion

**Figure 3:**
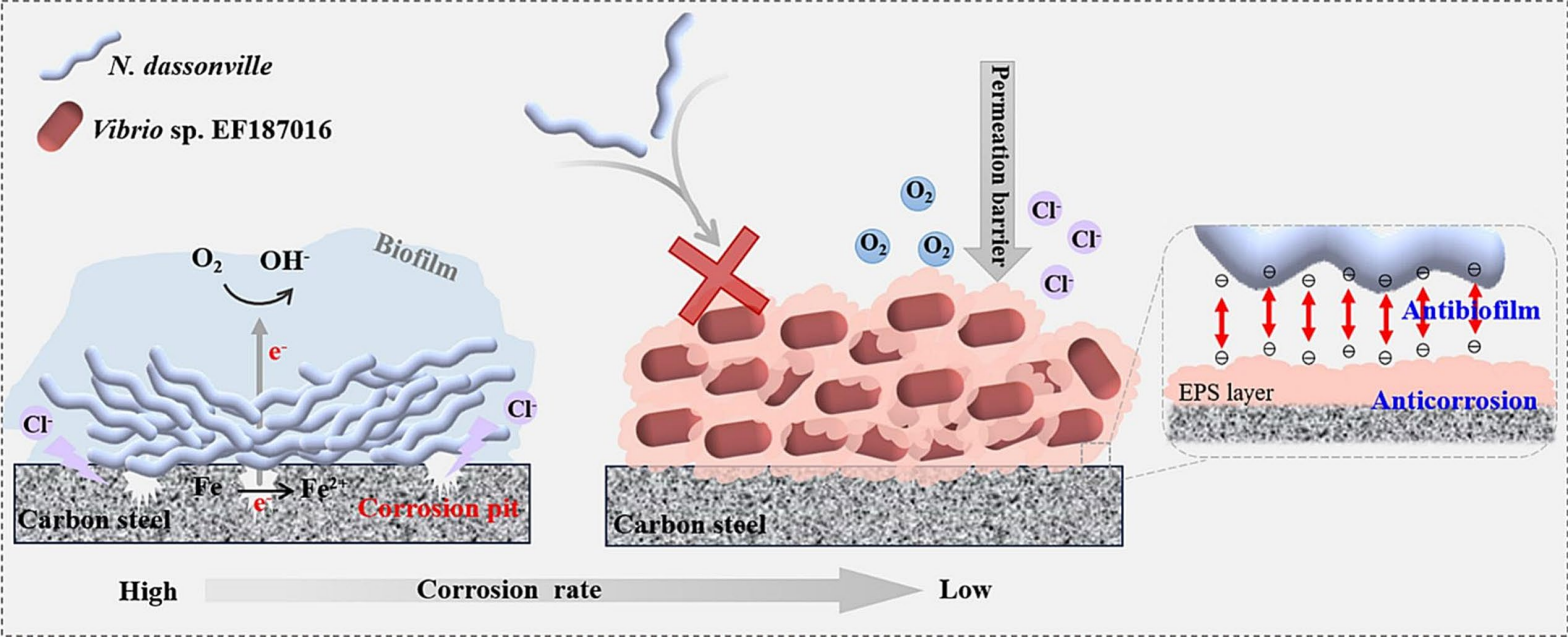
Biofilm of *Vibrio* sp. EF187016 inhibiting microbial corrosion caused by *Nocardiopsis dassonville*. Reprinted from Gao et al. (2024) with permission from Elsevier. The symbol X indicated that the attachment of *Nocardia dassonvillei* is hindered by the presence of extracellular polymeric substances (EPS) or a living biofilm.

With the rapid advancement of biofilm research, the dual role of biofilms in corrosion—both promoting and inhibiting corrosion—has gained increasing attention, highlighting the need to explore their corrosion-inhibiting potential further. Under aerobic conditions, aerobic and certain facultative microorganisms can inhibit metal corrosion by reducing the concentration of electron acceptors, such as oxygen, on the metal surface through their respiratory processes, thus limiting the electrochemical reactions that drive corrosion. This process impedes the cathodic depolarization thereby slowing down corrosion rates. San et al. (2014) discovered that dense biofilm formation by *Pseudomonas aeruginosa* can protect nickel-zinc alloys, and even facilitate the repair of damaged surfaces. Moradi et al. (2015) found that marine *Vibrio neocaledonicus* sp. can effectively inhibit the corrosion of carbon steel in seawater. The primary mechanism of corrosion inhibition is believed to be the consumption of oxygen through microbial respiration, while the uniform biofilm formed by the microorganisms (*Vibrio* sp. EF187016) serves as an effective shield, protecting the metal surface from corrosion caused by *Nocardiopsis dassonville* (Figure 3; Gao et al., 2024).

### 4.2 Secretion of antimicrobial substances and inhibition of metal corrosion

Antimicrobial surfactants can adsorb to metal surfaces thereby reducing cell adhesion (Zhu et al., 2021). They can also damage cell membranes by generating reactive oxygen species (ROS), which have dual effects: corrosion inhibition and bactericidal activity. The ROS can inhibit corrosion by reducing the availability of electron acceptors, while also killing harmful bacteria, thereby limiting microbial-induced corrosion (Tawfik et al., 2023). Purwasena et al. (2019) developed a novel antimicrobial agent using a biosurfactant produced by *Bacillus sphaericus* isolated from a primary oil reservoir. This biosurfactant efficiently inhibited the attachment of *Pseudomonas aeruginosa* to the surface of carbon steel ST37 and could also remove existing biofilm from the steel surface, thus reducing microbial corrosion.

Polyaspartic acid is one of the naturally-occurring polyamino acids and a new type of green scale and corrosion inhibitor that is non-toxic (Cui et al., 2011; Gao et al., 2015). It can form chelates with various ions, such as iron, copper, calcium, and magnesium, and adhere to the metal surface, thereby creating a protective layer that helps prevent metal corrosion. An engineered bacterium *Baciillus subtilis*, capable of secreting polyaspartate peptide, has been reported to reduce the corrosion of passive aluminum alloy at pH 6.5, likely through the formation of a protective layer that minimizes the interaction between the metal surface and corrosive agents (Örnek et al., 2002).

### 4.3 Biological competition inhibition of corrosion

**Figure 4:**
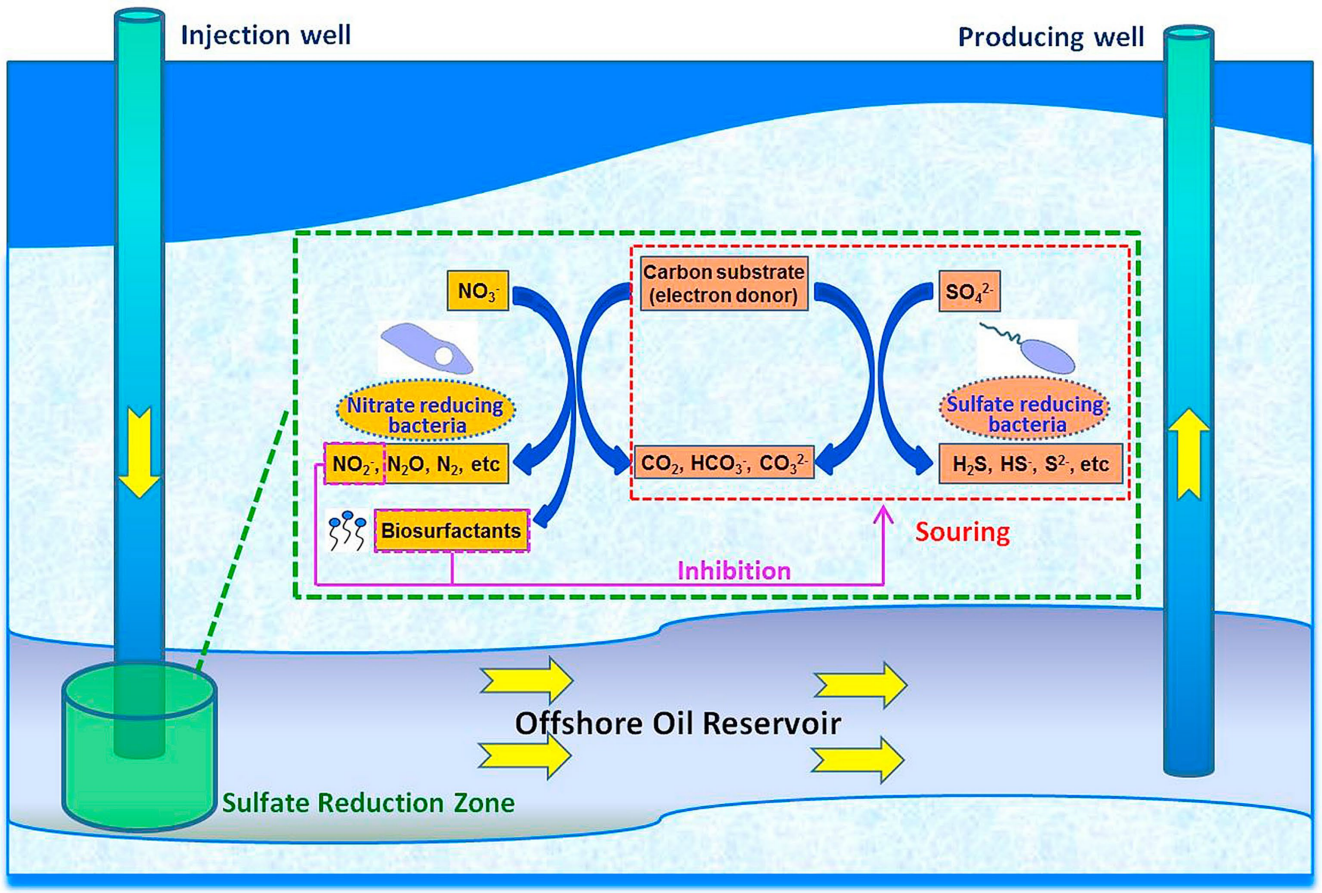
Biosurfactant involved NRB-SRB interactions in a souring reservoir system (Fan et al., 2020; Reprinted from Fan et al. (2020) with permission from Elsevier).

Inhibition of corrosion of metallic materials by employing the competitive relationship between organisms is an increasingly important method of microbial corrosion prevention. One approach for preventing SRB-induced corrosion in oilfields was to encourage the inhibitory relationship between SRB and nitrate-reducing bacteria (NRB). NRB can outcompete SRB for nutrients and space, thereby inhibiting their growth. Additionally, NRB can oxidize corrosive H₂S, therefore reducing corrosion (Figure 4; Fida et al., 2016; Fan et al., 2020). *E. coli* can produce acids that corrode metals but when coexisting with *Pseudomonas fluorescens*, the biofilm formed by *P. fluorescens* can isolate the corrosive acids produced by *E. coli*, the diffusion of dissolved oxygen on the metal surface and promotes the conversion of *α*-FeOOH and *γ*-FeOOH to Fe₂O₃ and Fe₃O₄, thereby slowing down the corrosion caused by *E. coli*. This process helps to stabilize the protective oxide layer and mitigate microbial-induced corrosion (Chu et al., 2020). Furthermore, it has been found that co-culture of planktonic sulfate-reducing bacteria (SRB) with iron-oxidizing bacteria (IOB) can inhibit the growth of SRB, thus reducing corrosion (Liu et al., 2015).

### 4.4 Biomineralization inhibition of metal corrosion

**Figure 5:**
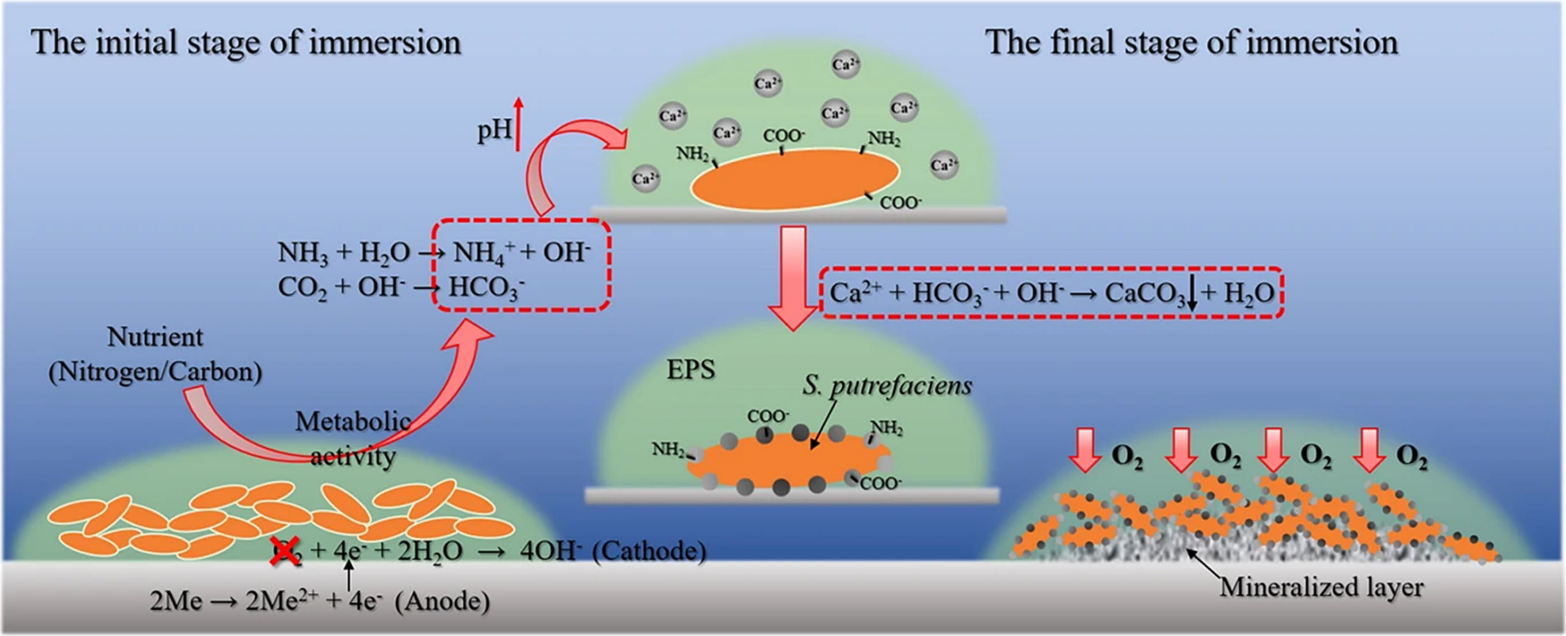
Microbiologically influenced corrosion inhibition by biomineralization mediated by *Shewanella putrefaciens* (Lou et al., 2021). Reproduced from Lou et al. (2021), licensed under CC BY 4.0.

Biomineralization refers to the process by which organisms deposit minerals, often involving the precipitation of minerals that contain both inorganic components and organic materials. This process plays a crucial role in the formation of structures like shells, bones, and some protective biofilms (Li et al., 2014; Li et al., 2016). Previous work has demonstrated that biomineralization provides a promising strategy for toxic metal immobilization and cement solidification (Li et al., 2016; Li et al., 2020a; Li et al., 2020b). In recent years, it has been found that biomineralization can also play an important role in inhibiting corrosion with biomineralization process involving extracellular polymeric substances (EPS) that bind surrounding metal ions, leading to the formation of solid minerals which inhibit metal corrosion by creating a barrier that blocks corrosive elements in the environment (Figure 5; Kip and Van Veen, 2015; Li et al., 2021; Lou et al., 2021). In the biomineralization process, the biofilm also plays an important role, and under certain specific conditions, it can transform pre-existing rust layers into dense, uniform biomineralized coatings, effectively reducing metal corrosion.

The number of biominerals related to microbial corrosion inhibition has increased greatly and includes more than 60 kinds of minerals, the predominant biominerals being calcium carbonates, calcium phosphates, silicon oxides, iron oxides and sulfates (Table 2).

**Table 2:** Typical microorganisms and the biominerals formed to inhibit corrosion.

Microorganisms	Biominerals	Mineral substrate	Reference
*Streptomyces* sp.	CaCO_3_, BaSO_4_	X65 steel	Wang et al. (2023)
*Bacillus mucilaginosus Krassilnikov*	CaCO_3_	Portland cement	Hu et al. (2024)
*Bacillus velezensis*	CaCO_3_	X65 steel	Shan et al. (2024)
*Shewanella putrefaciens*	CaCO_3_	Q235 steel	Lou et al. (2023)
Iron-oxidizing bacteria	Fe_2_O_3_	Q235 steel	Liu et al. (2016)
*Pseudomonas stutzeri*	Fe_3_O_4_, FeOOH	X80 steel	Liu et al. (2022)
*Pseudoalteromonas* sp.	Fe_3_O_4_	Steel sheet	Liu et al. (2018)
*Proteobacteria*, *Firmicutes*	Fe_2_O_3_, Fe_3_O_4_, Fe(OH)_2_	API 5 L 52 carbon steel	AlAbbas et al. (2013)
*Flamentous bacteria*	MnO_x_	UNSS30400 stainless steel	Kumar et al. (2020)
*Bacillus subtilis natto*	CaCO_3_	Concrete	Huynh et al. (2019)
*Desulfitobacterium hafniense*	Fe^2+^_3_(PO_4_)_2_·8H_2_O, Fe^2+^Fe^3+^_2_(PO_4_)_2_(OH)_2_	Steel plates	Comensoli et al. (2020)
*Exiguobacterium mexicanum*	CaCO_3_	Concrete	Bansal et al. (2016)

A biomineralization film formed by *Pseudomonas stutzeri*, isolated from seawater, was found to mainly compose nano Fe_3_O_4_ and FeOOH. The corrosion rate of X80 pipeline steel increased with a decrease in initial bacterial cell concentration but 10^7^ cell/mL could promote a thick biomineralization film (~146 m), which resulting in corrosion inhibition with a corrosion rate of 0.058 ± 0.008 mm/y (Liu et al., 2022). Microorganisms can also produce biomineralized deposits, such as phosphate and carbonate layers, which form natural protective barriers on metal surfaces, thereby mitigating the risk of corrosion. In a study on the corrosion behavior of *Bacillus subtilis* on 2A14 aluminum alloy in seawater, the results showed that a biomineralized film containing CaMg(CO₃)₂ formed on the alloy surface, effectively inhibiting seawater erosion (Shen et al., 2020). Some extracellular enzymes like proteases, alkaline phosphatase, and carbonic anhydrase, can alter the environmental pH and precipitate calcium (Ca) and magnesium (Mg) ions as carbonates and phosphates to reduce microbial corrosion (Wang et al., 2022). Qu et al. (2015) investigated the corrosion process of *Bacillus subtilis* in a nutrient-rich simulated seawater medium. Initially, *Bacillus subtilis* accelerated corrosion by producing organic acids, but with time, the formation of a dense biofilm slowed down the corrosion rate. Additionally, co-culturing of *Pseudomonas aeruginosa* with *Bacillus subtilis* lead to synergistic biomineralization, forming a dense and stable anticorrosive film on steel (Guo et al., 2021).

In soil or aqueous environments, microorganisms including *Pseudomonas* sp., *Bacillus subtilis*, and *Shewanella* sp. that have exhibit biomineralization capabilities, offering potential for innovative corrosion protection technologies (Hao et al., 2022). Compared with traditional organic protective coatings, super hydrophobic anti-corrosion coatings, and self-repairing anti-corrosion coatings, a corrosion inhibition method based on microbial biomineralization has the advantages of simplicity, cost-effective, and environmental sustainability. In challenging environments where maintenance and repair are difficult, biomineralization may offer a promising and innovative future approach for surface corrosion protection technology.

## 5 Conclusion

Microbial corrosion is an extremely common phenomenon. Microorganisms demonstrate varying degrees of corrosiveness depending on the type of material. Given the differences in growth and metabolic activity of distinct groups of microorganisms under varying environmental conditions, along with the complex interactions among microbial species, even the same microorganism may exhibit different corrosion mechanisms toward the same metal. This variability highlights the dynamic nature of microbial-induced corrosion (MIC) and the need for tailored approaches in managing corrosion in different environments. To better understand microbial corrosion mechanisms, it is essential to study the composition of corrosive microbial communities. Much research focusses on single microbial strains, but in real-world scenarios, multiple microorganisms generally coexist, interact, and influence each other. This complexity, along with challenges in simulating natural conditions in the laboratory, presents a significant hurdle for advancing research on microbial corrosion. While traditional anticorrosion methods such as corrosion-resistant materials and protective coatings are effective, they carry risks of environmental contamination. A more targeted, cost-efficient, and eco-friendly approach is desired. Microbiological strategies offer promising alternatives, such as the development of ‘green’ self-healing coatings and targeted microbial inhibition. Microbial inhibition of corrosion introduces novel avenues for corrosion prevention, utilizing processes like biomineralization and the application of microbial metabolites to suppress corrosion. Furthermore, self-repairing protective layers could be designed based on biomineralization principles. To enhance the stability and longevity of corrosion protection, corrosion inhibition technology could be integrated with other approaches, environmentally-friendly corrosion inhibitors, green coatings, advanced antibacterial materials, and nanoparticles, all of which offer sustainable solutions for mitigating corrosion while minimizing environmental impact. These approaches aim to enhance the longevity of materials and prevent microbial-induced corrosion in various industrial applications.
